# Friction and Tribocorrosion Behavior of Fe-Cr-B Alloys Fabricated via Extreme High-Speed Laser-Induction Hybrid Cladding

**DOI:** 10.3390/nano16201282

**Published:** 2026-10-10

**Authors:** Pengfei Sun, Xinghua Wang, Chenghui Hu, Yanming Wu, Dengzhi Wang, Zhen Li

**Affiliations:** 1 Luoyang Ship Material Research Institute, China State Shipbuilding Corporation Limited (CSSC), Luoyang 471023, China; 2 International Joint Laboratory of Advanced Bulk Nanomaterials for Innovative Applications, Harbin Engineering University, Harbin 150001, China; 3 School of Materials Science and Engineering, Dalian University of Technology, Dalian 116024, China; 4 Wuhan National Laboratory for Optoelectronics, Huazhong University of Science and Technology, Wuhan 430074, China

**Keywords:** extreme high-speed laser-induction hybrid cladding, Fe-Cr-B alloy, friction behavior, tribocorrosion behavior

## Abstract

Fe-Cr-B coatings were fabricated using laser-induction hybrid cladding and extreme high-speed laser-induction hybrid cladding (EHIC), and their microstructural evolution, dry friction performance, and tribocorrosion behavior were systematically investigated. The results indicate that the coatings consist of a matrix and eutectic region comprising M_2_B and martensite. With increasing scanning speed, the coarse dendritic matrix transforms into an equiaxed structure, and the overall microstructure becomes significantly refined. The dry friction mechanisms of the Fe-Cr-B coating involve oxidation and abrasive wear. The eutectic region constitutes the primary load-bearing and wear-resistant constituent within the coating. Increasing its fraction directly enhances the overall wear performance. Furthermore, the formation of a tribolayer during testing contributes to improved wear resistance. These combined factors lead to a reduction in the wear rate from 99.7 × 10^−3^ mm^3^·N^−1^·m^−1^ to 50.2 × 10^−3^ mm^3^·N^−1^·m^−1^ as the scanning speed increases. In corrosive media, the formation of a tribolayer is hindered, and electrochemical corrosion diminishes the resistance of the coating to plastic deformation, leading to a substantial increase in the wear rate. Under these conditions, the wear mechanism transitions to one dominated by abrasive wear. Owing to the high fraction of the eutectic region in the EHIC coating, its tribocorrosion performance is superior. As the scanning speed increases, the wear rate decreases from 255.8 × 10^−3^ mm^3^·N^−1^·m^−1^ to 175.4 × 10^−3^ mm^3^·N^−1^·m^−1^.

## 1. Introduction

Surface failure is the predominant cause of premature degradation and eventual scrapping of mechanical components operating under harsh service conditions [1,2,3]. The associated energy dissipation and material loss have become critical factors limiting the reliability and cost-effectiveness of high-end equipment. Consequently, the fabrication of high-performance protective coatings through advanced surface engineering techniques has emerged as a vital research direction in materials science and engineering [4,5,6], aiming to achieve a synergistic enhancement of wear resistance, corrosion resistance, and mechanical strength.

Unlike traditional iron-based alloys that rely on carbide strengthening, the Fe-Cr-B alloy leverages the in-situ precipitation of high-hardness borides to achieve exceptional macroscopic hardness and wear resistance without requiring a high carbon content [7,8,9]. Concurrently, the solid-solution effect of chromium confers enhanced corrosion resistance [10]. Consequently, Fe-Cr-B alloys hold significant promise for applications in corrosion-resistant wear coatings. Under tribocorrosion conditions, material degradation is governed by the interaction between mechanical wear and corrosion. For Fe-Cr-B alloys, such tribocorrosion behavior is closely related to their heterogeneous microstructure. In particular, the fraction and distribution of the hard M_2_B containing eutectic regions affect the load-bearing capacity and resistance to abrasive wear, whereas the matrix and matrix/boride interfaces are more susceptible to corrosion-assisted degradation. Previous studies have shown that the tribocorrosion behavior of Fe-Cr-B alloys is strongly dependent on the characteristics of boride-containing microstructures. Coarse or heterogeneously distributed borides may promote localized stress concentration and interfacial degradation, whereas refined and uniformly distributed M_2_B phases can provide more continuous load support and improve resistance to abrasive wear and corrosion-assisted material removal [7,8]. Therefore, the microstructural characteristics of the matrix and eutectic regions play an important role in determining the tribocorrosion resistance of Fe-Cr-B alloys. However, their high cracking susceptibility poses a major limitation for coating preparation [11].

Laser-induction hybrid cladding technology (LIC) [1,12,13] developed in recent years, mitigates this issue by preheating the substrate via induction heating either synchronously with or prior to laser irradiation, thereby effectively controlling molten pool solidification and thermal stress distribution. This approach combines the high precision of laser processing with the wide-area thermal effect of induction heating, reducing the temperature gradient and offering unique advantages for crack suppression. Nevertheless, as the induction heating temperature increases, the coating becomes susceptible to microstructural coarsening and consequent performance degradation [14].

In response to the drive toward higher efficiency and precision in surface engineering, ultra-high-speed laser cladding technology has emerged [15]. By optimizing the laser-powder coupling mechanism, this technique increases cladding efficiency by an order of magnitude and produces coatings with finer, more uniform microstructures and extremely low dilution rates [16,17,18]. However, the accompanying extremely high cooling rate significantly elevates internal stress levels within the coating, rendering it more prone to cracking [19].

Integrating induction heating with extremely high-speed laser cladding, extreme high-speed laser-induction hybrid cladding (EHIC) offers the potential to achieve high-efficiency fabrication of highly crack-sensitive alloys [20,21]. Furthermore, the assisted thermal field is expected to further regulate the microstructure and alleviate residual stresses, thereby fundamentally addressing the issue of cracking. However, the microstructural evolution induced by EHIC on the dry wear and tribocorrosion behavior of Fe-Cr-B coatings remains insufficiently understood.

In light of this, the present study utilizes EHIC technology to fabricate Fe-Cr-B alloy coatings. The microstructural evolution under varying processing parameters is investigated, and the tribological behavior and failure mechanisms are comparatively analyzed under both dry and corrosive conditions. The intrinsic relationship between microstructure and wear performance is elucidated. This work is expected to offer a novel technical approach and theoretical foundation for the development of highly dense, crack-free Fe-Cr-B alloy coatings with superior wear and corrosion resistance.

## 2. Experimental Procedures

### 2.1. Material Preparation and Coating Fabrication

In this study, Fe-Cr-B alloy powders with particle sizes ranging from 38 to 120 μm were used to fabricate coatings on AISI 5140 steel substrates. The chemical composition of the powders was Fe-14.26Cr-1.25B-0.37C (wt.%). Prior to fabrication, the powders were dried in a vacuum oven at 120 °C, and the substrate surfaces were mechanically polished to remove any surface contaminants.

Coating fabrication was performed using a self-development device equipped with induction heating, enabling simultaneous heating during the cladding process. Our previous work demonstrated that induction heating at 300 °C was sufficient to achieve crack-free Fe-Cr-B coatings [14]. Accordingly, the present study focuses on coatings prepared with induction heating at 300 °C. The optimal process parameters are listed in Table 1. Coating samples are designated by their scanning speed, denoted as *x*-300 (where *x* = 1, 10, 20, and 40 m/min).

### 2.2. Microstructural Characterization

Samples for microstructural characterization were ground using silicon carbide sandpaper from 400 to 2000 grit, polished with diamond suspension, and then etched with an iron chloride solution (5 g FeCl_3_, 10 mL HCl, and 90 mL absolute alcohol) for 5 s. For quantitative microstructural analysis, optical micrographs were randomly acquired from at least five different regions of each coating at a magnification of 100×. Image-Pro Plus 6.0 software was used to identify the primary matrix and eutectic regions based on contrast differences and to determine their area fractions. The average values obtained from the analyzed fields were used as the representative area fractions. A scanning electron microscope (SEM, ZEISS, Oberkochen, Germany) equipped with an electron probe microanalyzer (EPMA, Shimadzu, Kyoto, Japan) was employed to examine the microstructure and elemental distribution of the coatings. Further microstructural analysis was conducted using a field-emission transmission electron microscope (FETEM, Tecnai G2 F30, Thermo Fisher Scientific, Waltham, MA, USA).

### 2.3. Performance Tests

The microhardness of the coatings was measured on both the cross-section and top surface using a Vickers-1000 tester (Shandong Laishi Automation Technology Co., Ltd., Qingdao, China) under a load of 1 kg applied for 15 s. Prior to wear testing, all specimens were ground and polished to ensure a consistent surface finish. Dry friction tests were conducted using a 6 mm diameter Si_3_N_4_ ball as the counterbody, while tribocorrosion tests were performed in a 3.5 wt.% NaCl solution. The testing parameters included an applied load of 30 N, a reciprocating speed of 10 mm/s, a stroke length of 5 mm, and a total test duration of 240 min. The wear characteristics were characterized using a three-dimensional confocal laser scanning microscope (CLSM), a scanning electron microscope (SEM), and X-ray photoelectron spectroscopy (XPS). All the tests were conducted three times or more to ensure the accuracy of the results.

Nanoindentation tests were performed using an Anton Paar NHT3 nano-indenter (Anton Paar GmbH, Graz, Austria) to determine the nano-hardness and elastic modulus of the matrix and eutectic regions in the coatings. Prior to testing, the coating specimens were ground with abrasive papers and mechanically polished, followed by ultrasonic cleaning in alcohol and acetone to remove surface contaminants. The maximum applied load was 5 mN, and the indentation depth was 1000 nm. At least three valid indentations were performed for each microstructural region, and the average values were used for analysis.

## 3. Results

### 3.1. Microstructure

**Figure 1:**
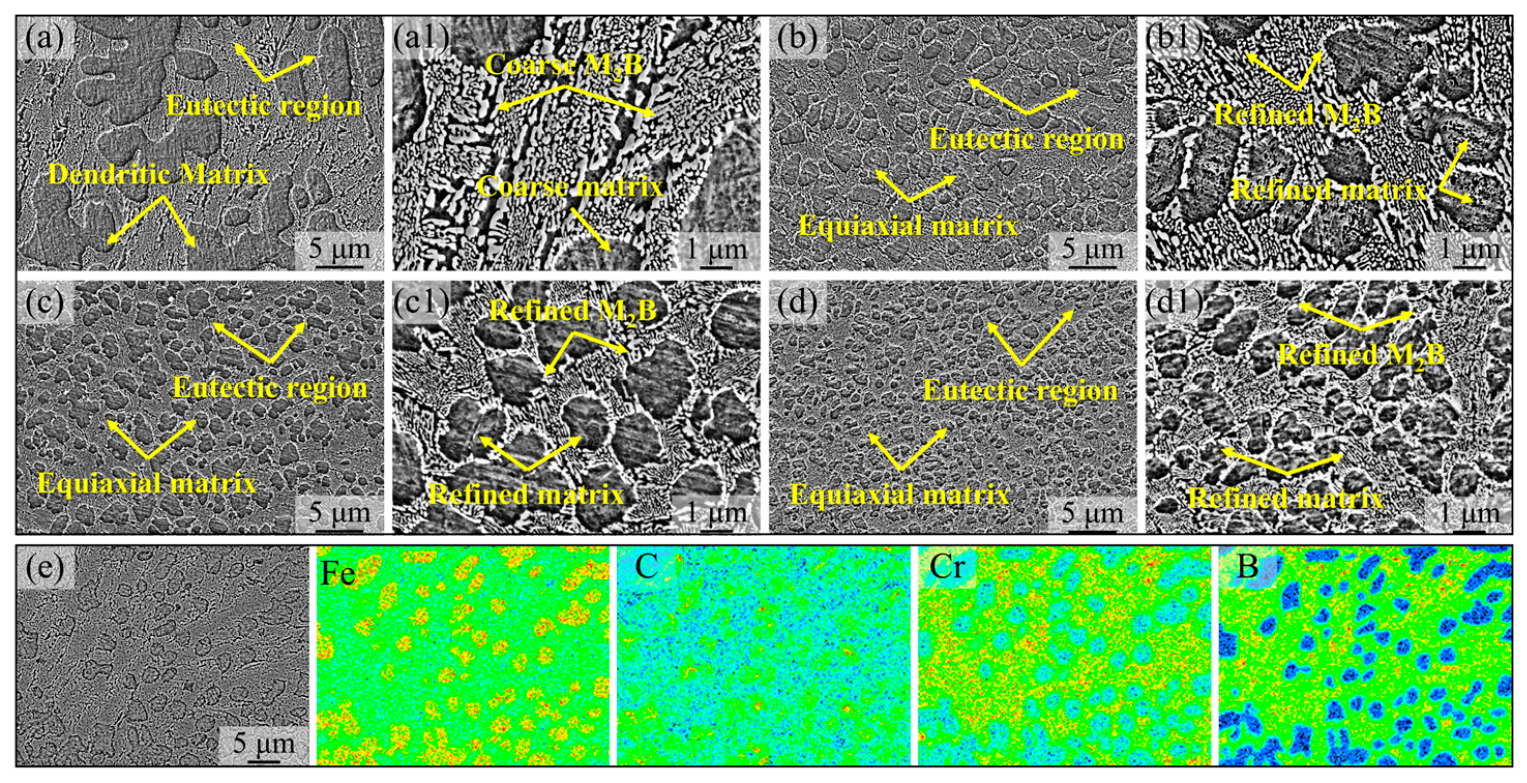
The microstructure of Fe-Cr-B coatings fabricated by LIC and EHIC: (**a**) LIC, (**b**) EHIC1, (**c**) EHIC2, (**d**) EHIC3, (**a1**–**d1**) high-magnification images of a-d, (**e**) EPMA mappings.

Figure 1 presents the microstructure of the Fe-Cr-B coatings fabricated at different scanning speeds. As shown in Figure 1a–d, the microstructure of each coating consists of a matrix and eutectic region. For the LIC coating (V = 1 m/min), the matrix exhibits coarse columnar or dendritic morphology. As the scanning speed progressively increases to 40 m/min, the microstructure of the coating is refined, with the matrix transforming into fine equiaxed or columnar structures, leading to a marked improvement in overall microstructural uniformity.

As shown in Figure 1a1–d1, the eutectic region within coatings comprises a bright M_2_B phase and darker alternating regions. At a scanning speed of 1 m/min, the M_2_B phase appears as coarse fishbone-like structures and short rods. With increasing scanning speed, the coarse M_2_B morphologies disappear. Both the size of the M_2_B phase and the interphase spacing decrease substantially, and the morphology transitions from fishbone-like to a lamellar arrangement. The EPMA mappings reveal that the matrix is enriched in Fe and C elements, while the eutectic region is composed of Cr and B elements.

**Figure 2:**
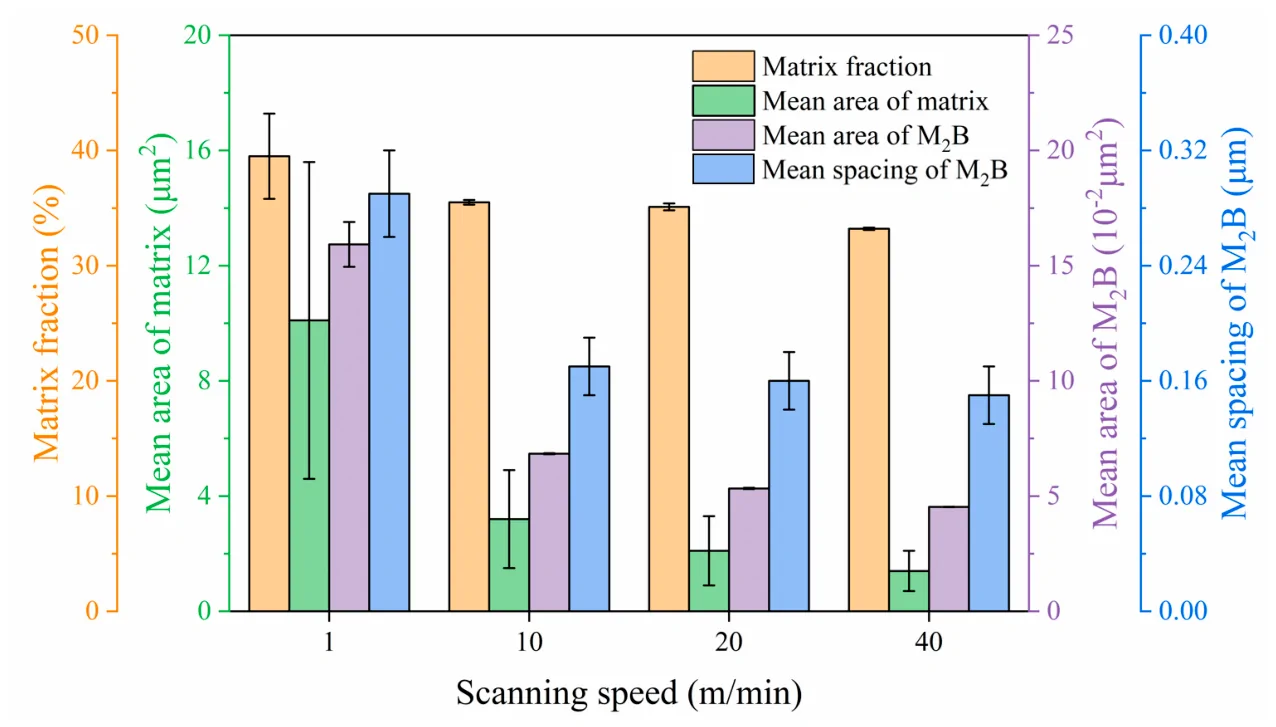
The microstructure features of coatings fabricated with different scanning speeds.

Figure 2 shows microstructural features for coatings fabricated at different scanning speeds, including the fraction and mean area of matrix, as well as the mean area and spacing of the M_2_B phase within the eutectic region. The results indicate that all features decrease as the scanning speed increases from 1 m/min to 40 m/min. Specifically, the matrix fraction declines from 39.5% to 33.2%, and its mean area decreases from 10.1 μm^2^ to 1.4 μm^2^. Meanwhile, the mean area of the M_2_B phase within the eutectic region diminishes from 15.9 × 10^−2^ μm^2^ to 4.5 × 10^−2^ μm^2^, and the M_2_B phase spacing narrows from 0.29 μm to 0.15 μm.

From Fe-B phase diagram [22], the matrix preferentially precipitates from liquid phase as temperature of molten pool decreases to 1174 °C. The solidification and cooling rates of the molten pool increase as the scanning speed increases [23], leading to greater undercooling, which promotes nucleation of the matrix and limits its coarsening. Meanwhile, the growth of eutectic region depends on atomic diffusion [24]. The increasing solidification and cooling rate also limit atomic diffusion, resulting in decreasing size and spacing of M_2_B phase.

**Figure 3:**
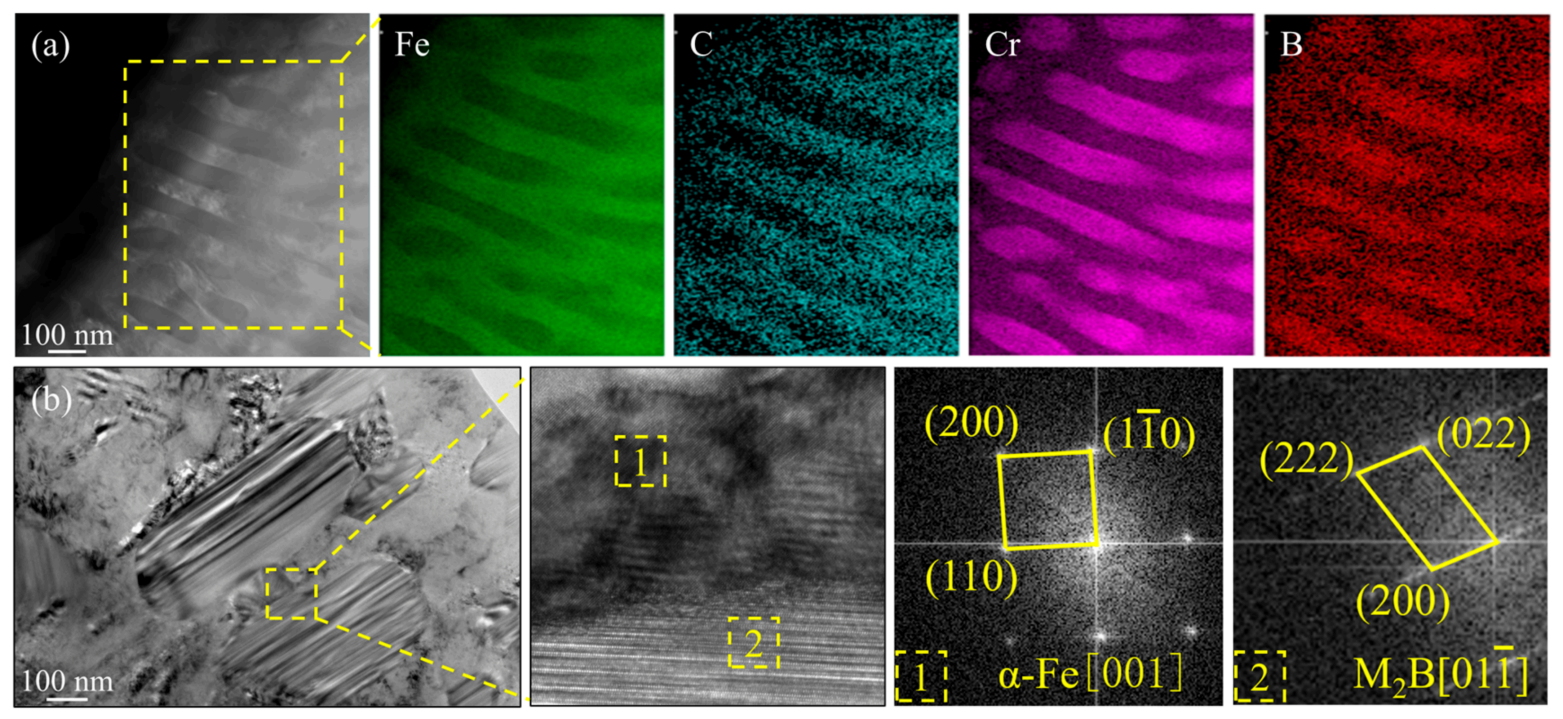
TEM results of the eutectic region within the Fe-Cr-B coating: (**a**) TEM-EDX results, (**b**) BF-TEM images and SAEDs of different regions.

Figure 3 shows TEM results of eutectic region within Fe-Cr-B coating. From TEM-EDX results, Cr and B are predominantly concentrated in the particulate phase, whereas Fe and C are primarily located in the surrounding interphase region. This elemental distribution confirms that the particulate phase corresponds to a Cr-rich boride, while the interphase region consists of an Fe-based solid solution.

From Figure 3b, obvious stacking fault features can be observed on the surface of the borides. Diffraction pattern analysis confirms that the eutectic region consists of M_2_B-type boride and bcc-Fe (α-Fe). The α-Fe may include both ferrite and martensite; their similar crystal structures preclude definitive differentiation by electron diffraction alone. However, ferrite contains extremely low carbon, whereas the Fe-based solid solution in the eutectic region exhibits carbon enrichment, as shown in Figure 3a, indicating that the interphase regions consist of martensite.

According to the literature [25], Fe_2_B possesses a body-centered tetragonal structure (a = b = 5.1317 Å, c = 8.5321 Å), while Cr_2_B exhibits a body-centered orthorhombic structure (a = 7.409 Å, b = 14.7118 Å, c = 4.2498 Å). The crystal structure of the M_2_B-type boride depends on the relative fractions of Fe and Cr atoms. M_2_B phases with high Fe content tend to adopt a structure similar to Fe_2_B, whereas those enriched in Cr more closely resemble the Cr_2_B structure. Goldfarb et al. [26] reported that Cr-rich M_2_B phases exhibit greater thermodynamic stability at elevated temperatures. During solidification, Cr atoms in the liquid phase substitute for Fe atoms in the Fe_2_B structure, reducing the free energy of the system. This atomic substitution, accompanied by the associated crystal structure transformation, readily induces stacking faults within the M_2_B phase.

### 3.2. Microhardness Tests

**Figure 4:**
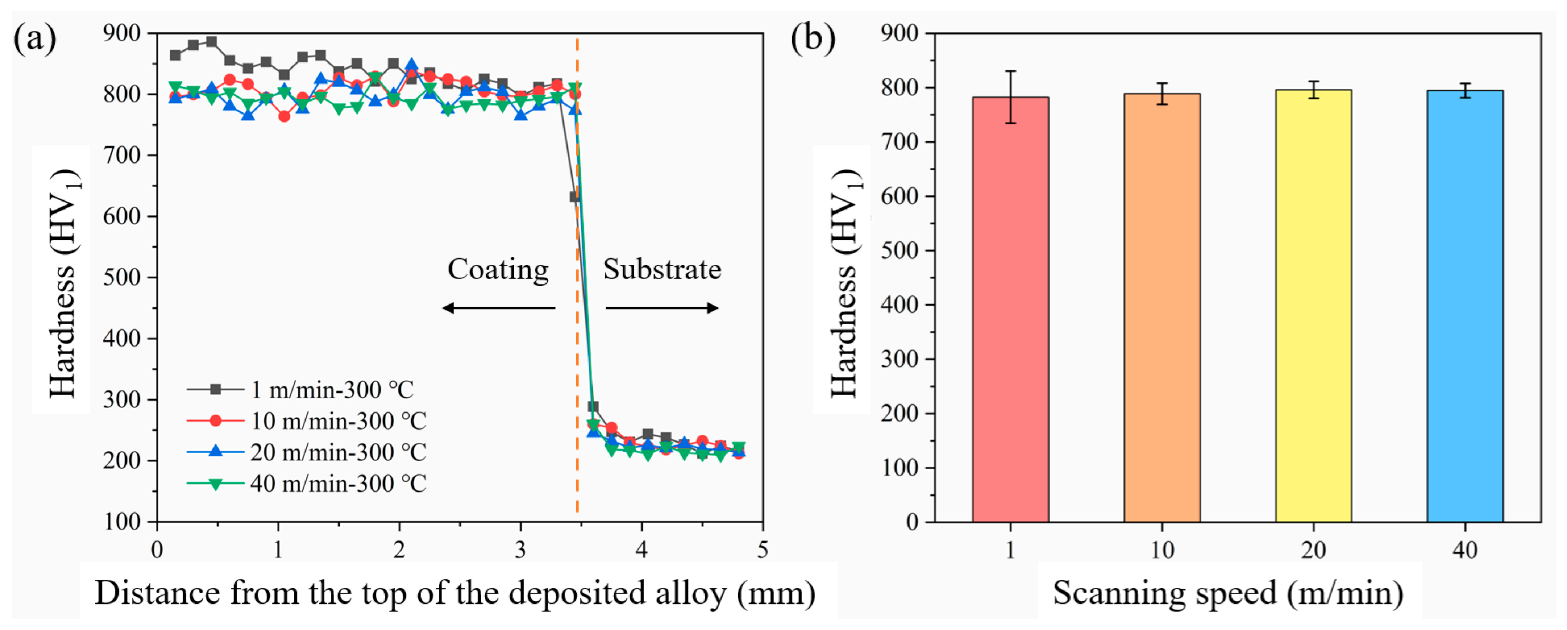
The microhardness of Fe-Cr-B coatings fabricated by LIC and EHIC: (**a**) depth-directional microhardness distribution, (**b**) surface average microhardness.

Figure 4 presents the microhardness test results for the Fe-Cr-B coatings fabricated by LIC and EHIC. As shown in Figure 4a, the microhardness of the LIC coating decreases from 863.6 HV_1_ at the surface to approximately 631.9 HV_1_ near the coating/substrate interface, representing a reduction of 26.8% along the depth direction. In contrast, the EHIC coatings exhibit a uniform microhardness distribution with depth, demonstrating significantly greater distribution stability than the LIC coating.

The average surface microhardness of each coating is shown in Figure 4b. The LIC coating has an average microhardness of 782.3 ± 48.8 HV_1_, and this considerable fluctuation indicates poor distribution uniformity. For the EHIC coatings, as the scanning speed increases, the average microhardness values are 789.6 ± 19.6 HV_1_ (10 m/min), 796.1 ± 16.6 HV_1_ (20 m/min), and 794.4 ± 13.2 HV_1_ (40 m/min), respectively, with a corresponding improvement in distribution uniformity. These results demonstrate that the EHIC technology enhances both the uniformity and stability of the hardness distribution in Fe-Cr-B coatings.

### 3.3. Dry Friction Behavior

**Figure 5:**
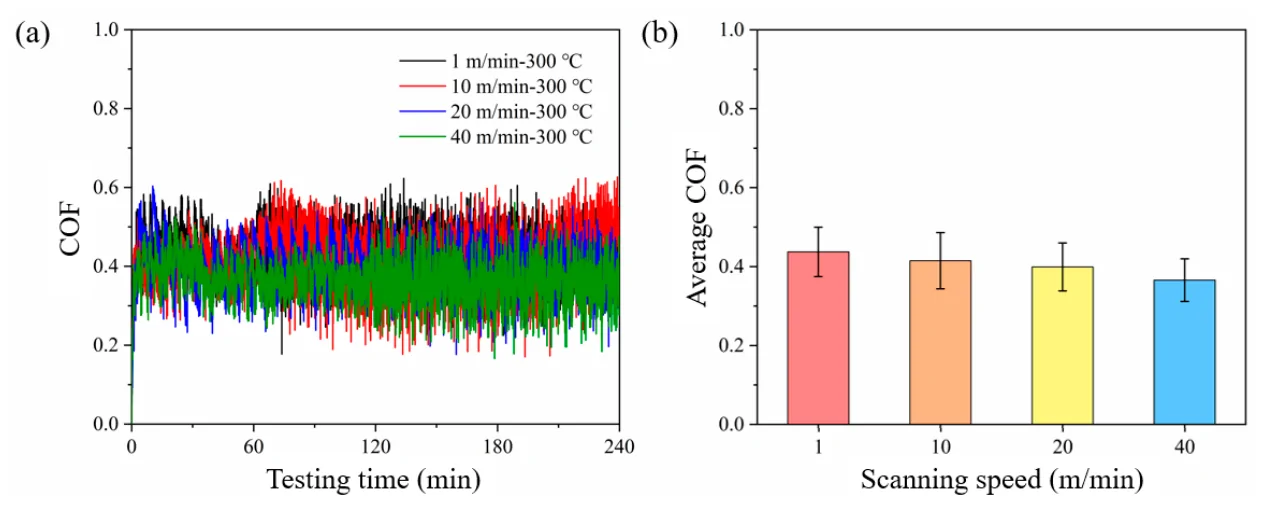
The COF of Fe-Cr-B alloys fabricated by LIC and EHIC in dry friction tests: (**a**) COF curves, (**b**) average COF values.

Figure 5 shows the coefficient of friction (COF) for dry friction of all Fe-Cr-B coatings. Figure 5a indicates that the COF curves of each coating exhibit considerable fluctuation throughout the test. The amplitude of these fluctuations gradually decreases with increasing scanning speed. The EHIC coating fabricated at 40 m/min displays the most stable COF curve, with the smallest fluctuation range. The average COF values, presented in Figure 5b, decrease from 0.44 to 0.37 as the scanning speed increases from 1 m/min to 40 m/min.

During the testing process, the COF is positively correlated with the actual contact area at the microscale [27]. The softer materials are more susceptible to plastic deformation, which increases the real contact area [28]. Within the Fe-Cr-B coating, the eutectic region containing the M_2_B phase exhibits greater resistance to plastic deformation than the matrix. During the reciprocating sliding process, the Si_3_N_4_ ball contacts this alternating hard-soft microstructure, causing dynamic variations in the contact area and consequently significant fluctuations in the COF. With increasing scanning speed, the microstructural uniformity of the coating improves, and the fraction of the eutectic region increases. This enhances the overall load-bearing capacity, suppresses the tendency for plastic deformation, and reduces the amplitude of COF fluctuations. These combined effects lead to the observed decrease in the average COF.

**Figure 6:**
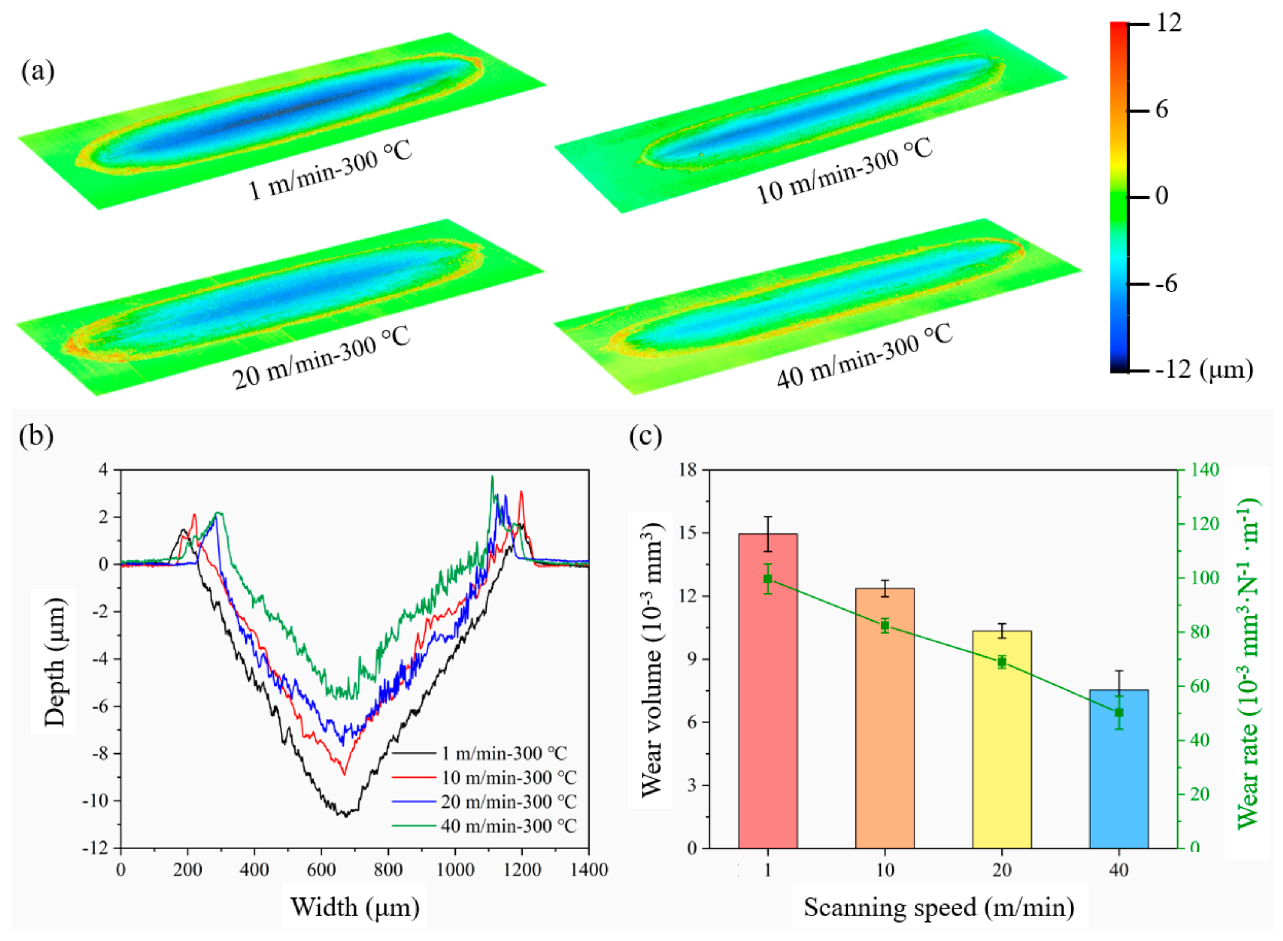
The wear characteristics of LIC- and EHIC-fabricated Fe-Cr-B alloys: (**a**) 3D morphology of the wear tracks, (**b**) 2D cross-section profiles of the wear tracks, (**c**) wear volume and wear rate.

Figure 6 presents the wear characteristics of the Fe-Cr-B coatings, with corresponding quantitative data provided in Table 2. The wear tracks all exhibit a typical V-shaped groove profile, surrounded by accumulated wear debris. As the scanning speed increases, the depth of the wear track gradually diminishes. The LIC coating exhibits the widest track and the largest wear volume (14.6 ± 0.8 × 10^−3^ mm^3^) and wear rate (99.7 ± 5.5 × 10^−3^ mm^3^·N^−1^·m^−1^). With increasing laser scanning speed, all measured wear characteristics show a decreasing trend, indicating a continuous enhancement in wear resistance. The EHIC coating fabricated at 40 m/min demonstrates the optimal wear resistance, with its wear volume and wear rate reduced to 7.5 ± 0.9 × 10^−3^ mm^3^ and 50.2 ± 6.1 × 10^−3^ mm^3^·N^−1^·m^−1^, respectively.

**Figure 7:**
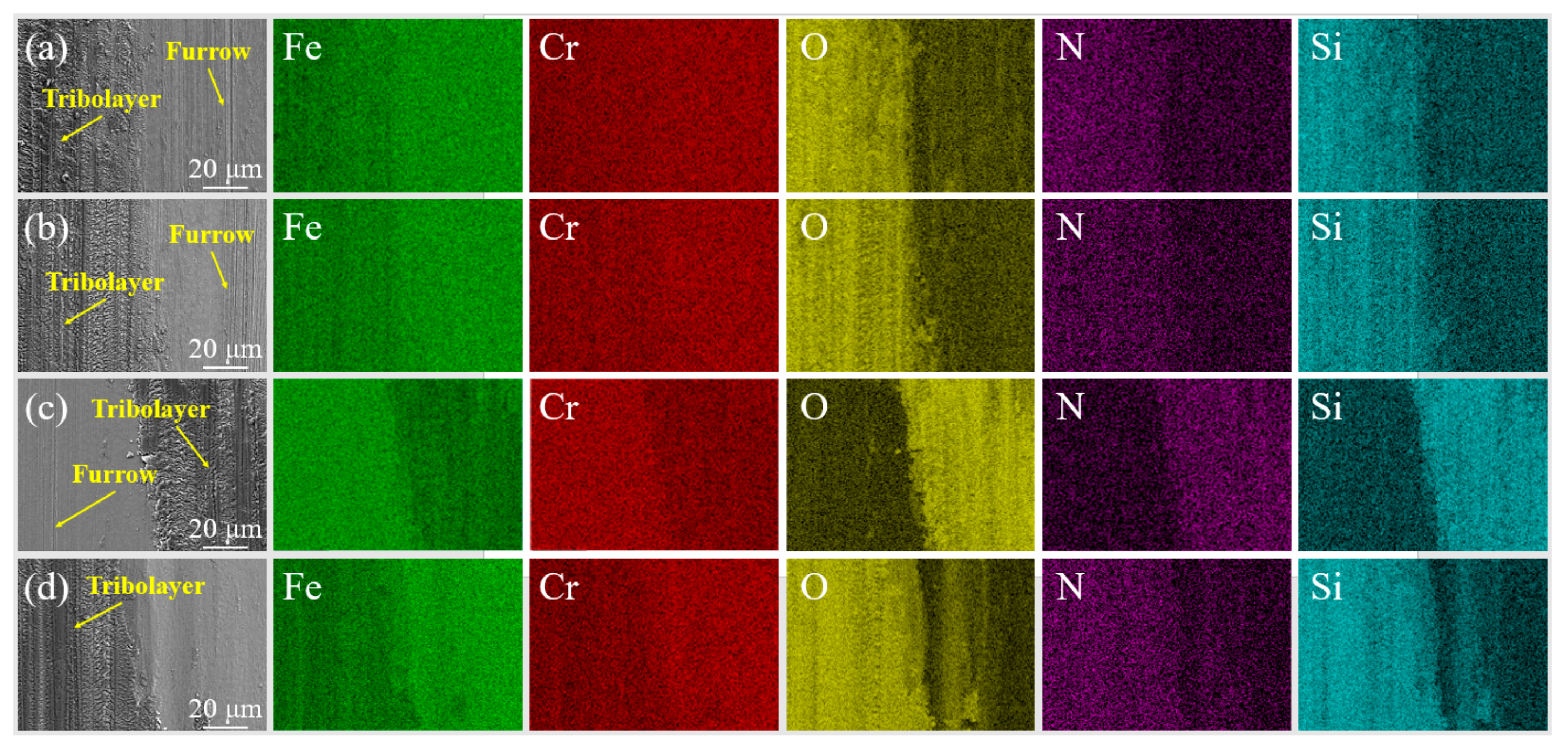
Wear morphology and elemental distribution of the coatings after wear test: (**a**) LIC, (**b**) EHIC1, (**c**) EHIC2, (**d**) EHIC3.

Figure 7 presents the wear morphology and elemental distribution of the coatings. The worn surfaces exhibit layered adhesive features and ploughing furrows. As the scanning speed increases, these furrows become progressively shallower, while the characteristics of the tribolayer remain largely unchanged. The elemental distribution analysis indicates that the wear process induces surface oxidation, with the tribolayer showing significant enrichment in O, N, and Si.

**Figure 8:**
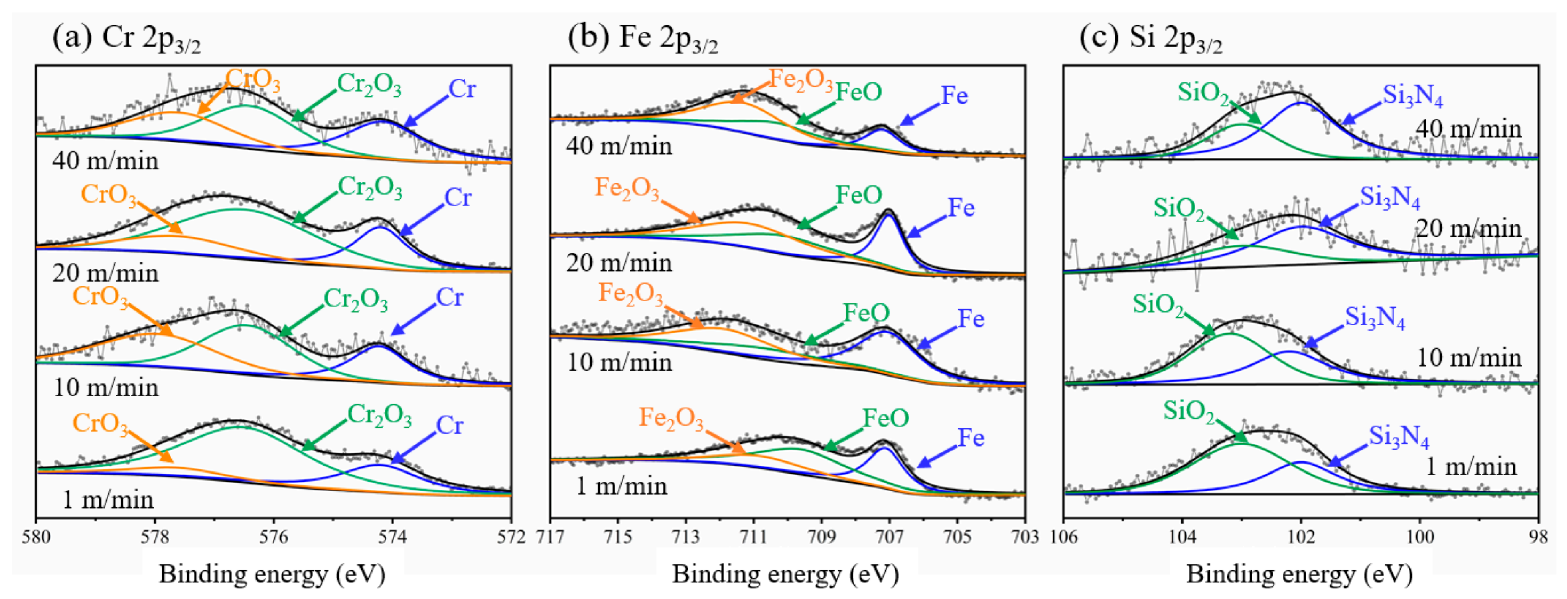
XPS spectra of worn surface of coatings after wear test: (**a**) Cr 2p_3/2_, (**b**) Fe 2p_3/2_, (**c**) Si 2p_3/2_.

Figure 8 presents the XPS spectra obtained from the wear surface of the coating. The Cr 2p_3/2_ spectrum shows characteristic peaks corresponding to Cr_2_O_3_ and CrO_3_. Similarly, the Fe 2p_3/2_ spectrum reveals the coexistence of FeO and Fe_2_O_3_. The Si 2p_3/2_ spectrum contains signatures of Si_3_N_4_ and SiO_2_, with the SiO_2_ attributed to the oxidation of Si_3_N_4_ abrasive particles induced by flash heating during the wear process [29]. Consequently, the tribolayer formed on the Fe-Cr-B coating surface is primarily composed of Cr_2_O_3_, CrO_3_, FeO, Fe_2_O_3_, SiO_2_, and Si_3_N_4_. This layer acts as a barrier that isolates direct contact between the Si_3_N_4_ abrasive ball and the coating, representing a principal mechanism for the excellent wear resistance of coating. In summary, the dominant dry friction mechanisms for the Fe-Cr-B coating are abrasive wear and oxidative wear.

### 3.4. Tribocorrosion Behavior

**Figure 9:**
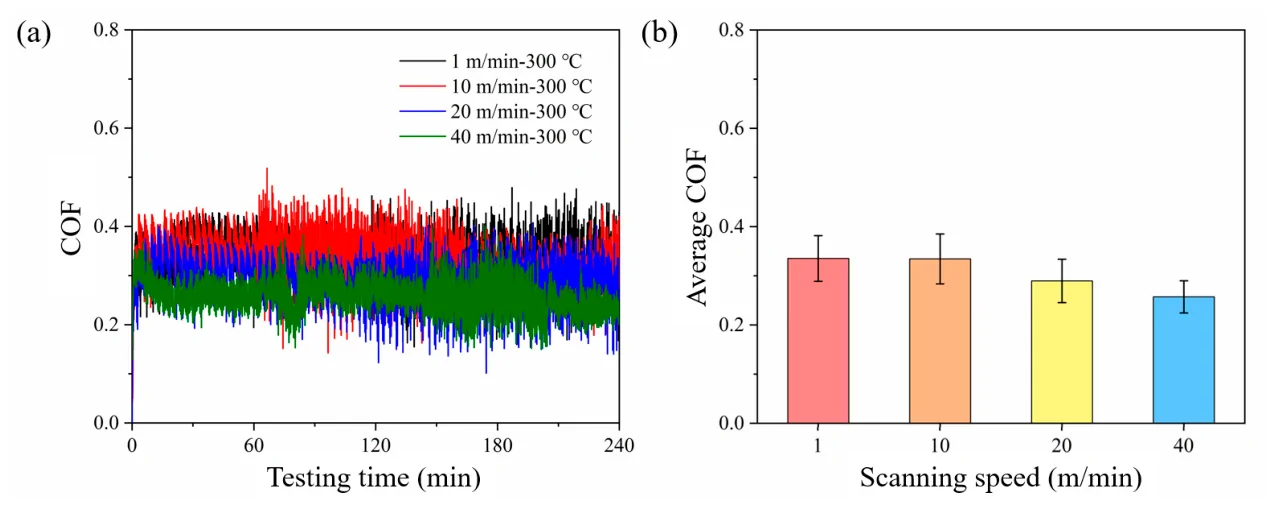
The COF of Fe-Cr-B alloys fabricated by LIC and EHIC in tribocorrosion tests: (**a**) COF curves, (**b**) average COF values.

Figure 9 presents the tribocorrosion COF of the Fe-Cr-B coatings fabricated using LIC and EHIC under 3.5 wt.% NaCl solution. Compared to the dry friction tests, the COF curves in the corrosive environment are lower for all coatings, although the overall decreasing trend with increasing scanning speed remained consistent. The LIC coating exhibited the highest COF values, which gradually decreased as the scanning speed increased. The corrosive solution contributes to friction reduction by dissipating frictional heat, thereby mitigating surface softening and adhesion tendencies while providing a lubricating effect. Furthermore, electrochemical reactions occurring in this environment can generate corrosion products on the coating surface. These products form a protective layer that isolates direct contact between the Si_3_N_4_ abrasive ball and the coating and provides additional lubrication, collectively leading to a further reduction in the COF [30].

**Figure 10:**
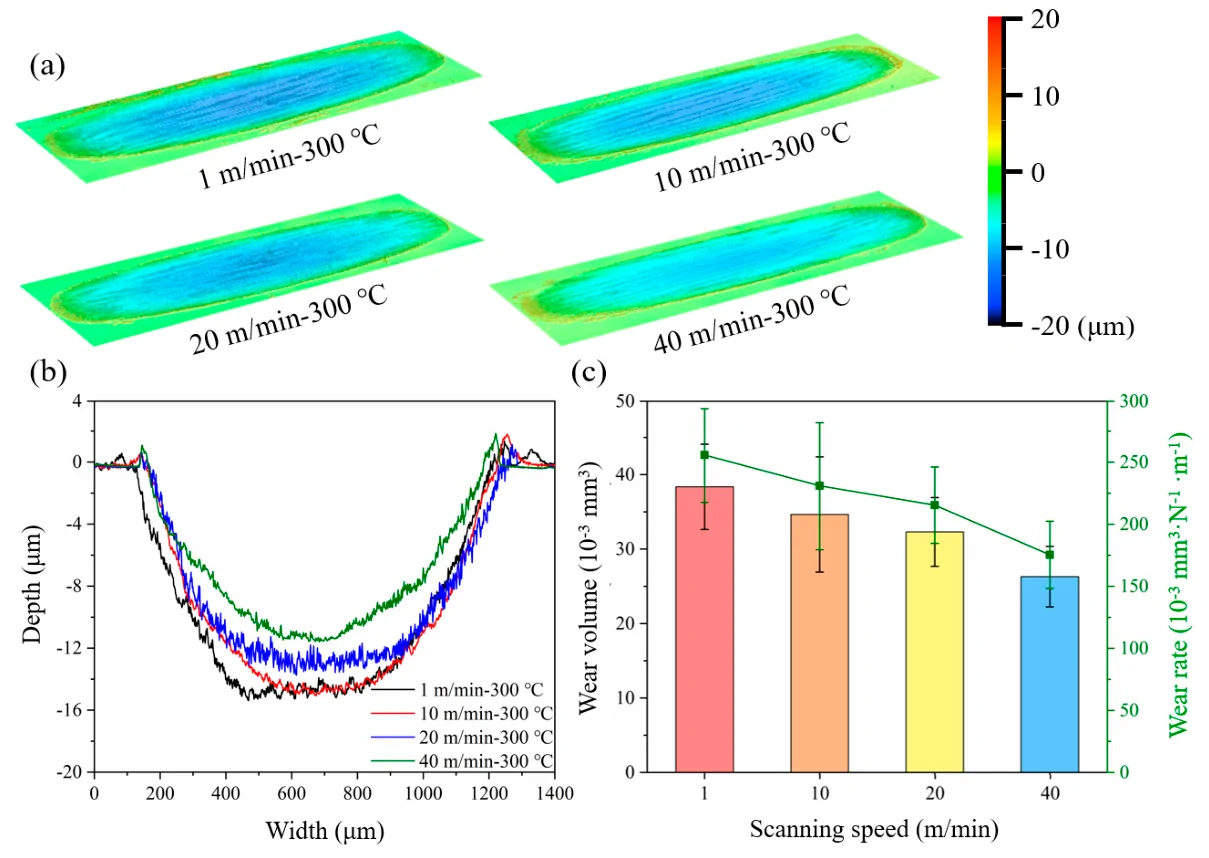
Tribocorrosion characteristics of LIC and EHIC fabricated coatings: (**a**) 3D morphology of the wear tracks, (**b**) 2D cross-section profiles of the wear tracks, (**c**) wear volume and wear rate.

Figure 10 presents the tribocorrosion characteristics of Fe-Cr-B coatings, with corresponding quantitative data provided in Table 3. As shown in Figure 10a, no pronounced debris accumulation layer is observed at the tribocorrosion crack. This absence is attributed to the NaCl solution environment, where generated debris is carried away by the reciprocating motion, preventing significant accumulation. Compared to dry friction, the tribocorrosion cracks exhibit greater width and depth, leading to a substantial increase in both wear volume and wear rate. The LIC coating demonstrated the poorest tribocorrosion resistance, with the largest wear volume (38.4 ± 5.7 × 10^−3^ mm^3^) and wear rate (255.8 ± 38.2 × 10^−3^ mm^3^·N^−1^·m^−1^). These values represent increases of approximately 263% and 256%, respectively, relative to the dry condition. When the scanning speed was increased to 40 m/min, the wear scar dimensions decreased. The corresponding wear volume and wear rate were reduced to 26.3 ± 4.1 × 10^−3^ mm^3^ and 175.4 ± 27.1 × 10^−3^ mm^3^·N^−1^·m^−1^. This constitutes an approximate 32% reduction compared to the LIC coating, yet remains approximately 350% higher than the values measured under dry friction. These results confirm that the corrosive environment significantly degrades the wear resistance of the Fe-Cr-B coatings.

**Figure 11:**
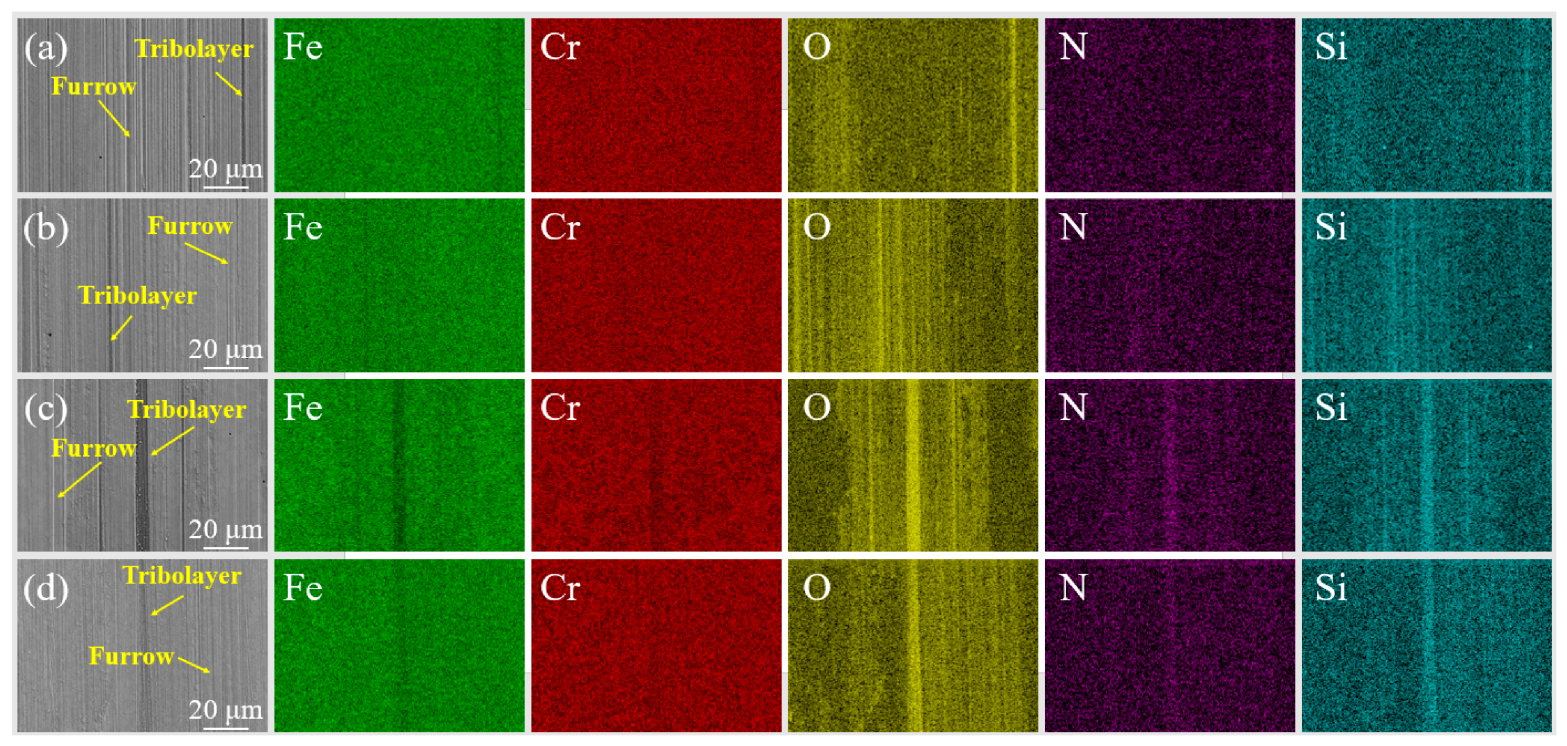
Wear morphology and elemental distribution of the coatings after tribocorrosion test: (**a**) LIC, (**b**) EHIC1, (**c**) EHIC2, (**d**) EHIC3.

Figure 11 presents the tribocorrosion morphology and elemental distribution of the coatings. Compared to the dry condition, the tribocorrosion morphology exhibits a greater number of deeper ploughing furrows and a significantly reduced tribolayer. The surface of the LIC coating is almost entirely covered by dense, deep furrows, with a tribolayer width of less than 5 μm. As the scanning speed increases, the severity of grooving gradually diminishes. For the coating fabricated at 40 m/min, only very shallow furrows and a limited tribolayer are observable. These results indicate that abrasive wear is the dominant mechanism for the Fe-Cr-B coatings under corrosive wear conditions.

**Figure 12:**
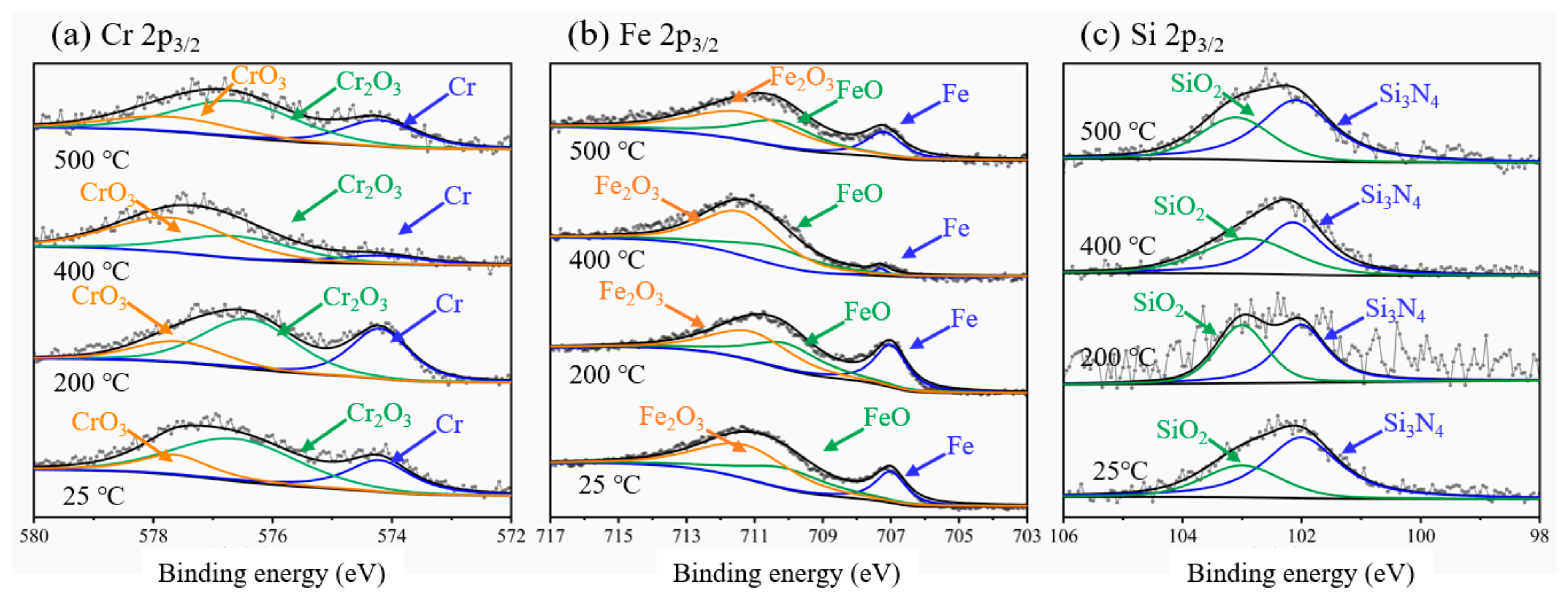
XPS spectra of worn surface of coatings after tribocorrosion test: (**a**) Cr 2p_3/2_, (**b**) Fe 2p_3/2_, (**c**) Si 2p_3/2_.

The elemental distribution within the wear scars reveals that the tribolayers formed under corrosive conditions contain the same primary elements (O, N, and Si) as those observed under dry friction. A comparison of morphology with elemental maps further shows that even regions without tribolayers exhibit significant oxygen enrichment. This indicates that pervasive electrochemical corrosion occurred across the sample surface during the tribocorrosion process. XPS analysis (Figure 12) confirms the presence of Cr_2_O_3_, CrO_3_, FeO, Fe_2_O_3_, SiO_2_, and Si_3_N_4_ within the wear scars.

Therefore, for the Fe-Cr-B coating, material loss during tribocorrosion is primarily dominated by abrasion resulting from continuous surface corrosion.

## 4. Discussion

### 4.1. Dry Friction Behavior of Fe-Cr-B

The tribological behavior and wear resistance of the material are critically influenced by its microstructure. For Fe-Cr-B coatings, the composite structure composed of the matrix and eutectic region directly governs its performance. Consequently, the micromechanical properties of the matrix and eutectic region were characterized, elucidating the correlation between these microstructural features and the wear performance of the coatings.

**Figure 13:**
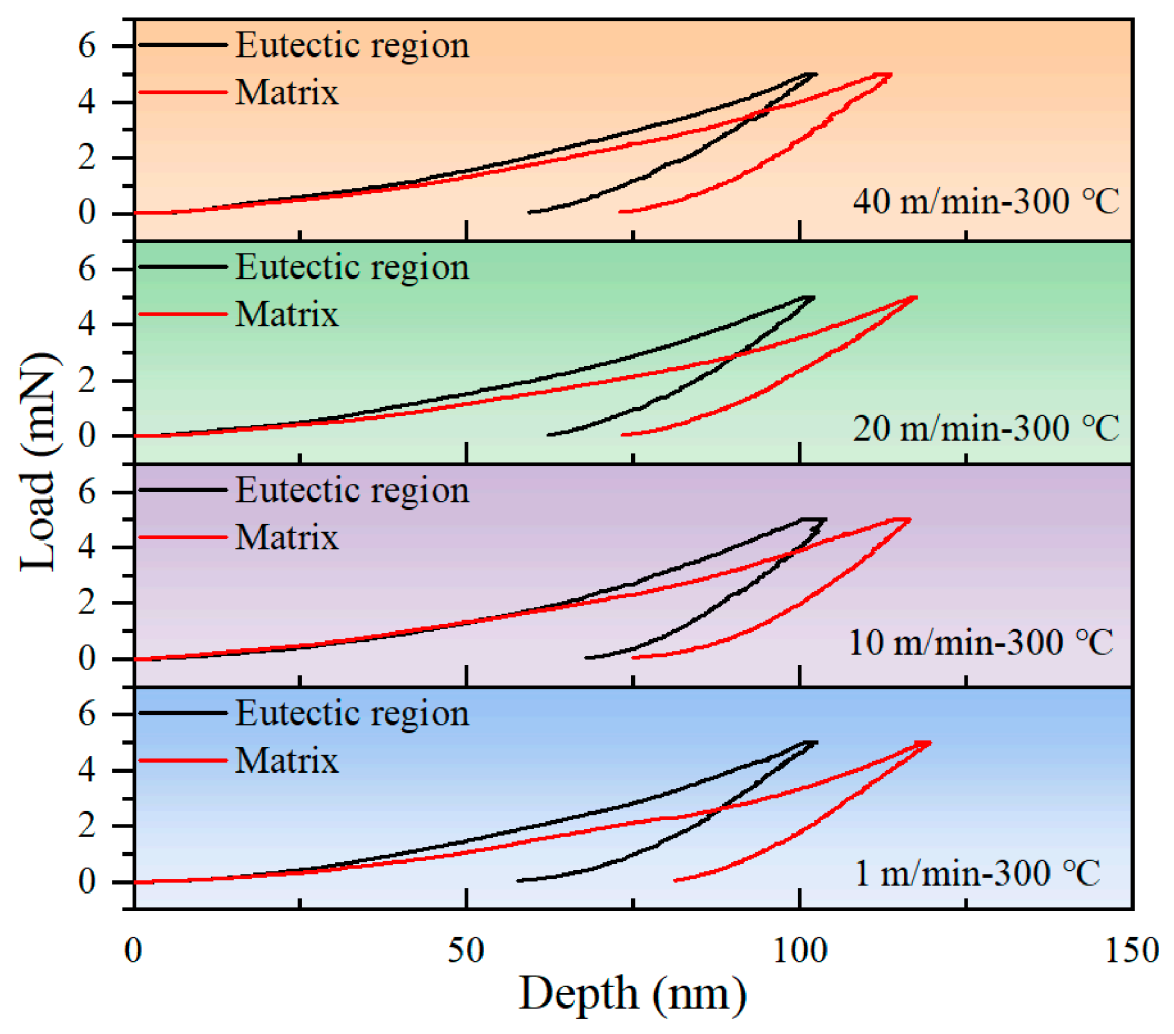
Load–displacement curves of different regions within each coating.

Figure 13 presents the nanoindentation load–displacement curves for the matrix and eutectic regions of each coating. The corresponding nano-hardness (H) and elastic modulus (E) values are listed in Table 4. The results demonstrate that for both regions in all coatings, the H and E values generally increase with increasing scanning speed. Notably, the eutectic region consistently exhibits higher H and E values than the matrix under all processing conditions.

The ratios H/E and H^3^/E^2^ are key indicators for evaluating a material’s load-bearing capacity and its resistance to plastic deformation, with higher values corresponding to superior wear resistance [6,31,32]. Data in Table 4 confirm that the eutectic region possesses higher H/E and H^3^/E^2^ values, establishing it as the primary load-bearing and wear-resistant constituent within the coating.

Archard [33] and Rabinowicz [34] proposed that wear performance is directly proportional to the applied load and inversely proportional to material hardness, underscoring hardness as a key determinant of wear resistance. However, in this study, Fe-Cr-B coatings exhibit significant variations in wear performance despite having comparable hardness and undergoing the same wear mechanisms. This discrepancy is attributed to the composite microstructure of the coating, which consists of a softer matrix and a harder eutectic region. The mismatch in mechanical properties between these regions gives rise to distinct local tribological responses during wear. Consequently, the wear behavior deviates from that of a homogeneous material.

According to Zum-Gahr [35], the wear behavior of a composite material results from the combined effect of its constituent phases. A wear model based on the rule of mixtures was proposed:

W_C_ = V_M1_W_M1_ + V_M2_W_M2_ (1)

where W_C_ is the total wear rate of the composite, V_M1_ and V_M2_ are the fractions of the matrix and reinforcing phases, respectively, and W_M1_ and W_M2_ are their corresponding wear rates. This model indicates that, provided the wear performance of each phase is stable, the overall wear resistance of the composite is linearly proportional to the fraction of the hard reinforcing phase.

The nanoindentation results indicate that the eutectic region in the Fe-Cr-B coating acts as the reinforcing phase. Its fraction rises with increasing scanning speed. Concurrently, the intrinsic wear resistance of both the matrix and eutectic regions is enhanced under these processing conditions. Collectively, these factors contribute to a progressive improvement in the overall wear resistance of the coating as the scanning speed increases.

**Figure 14:**
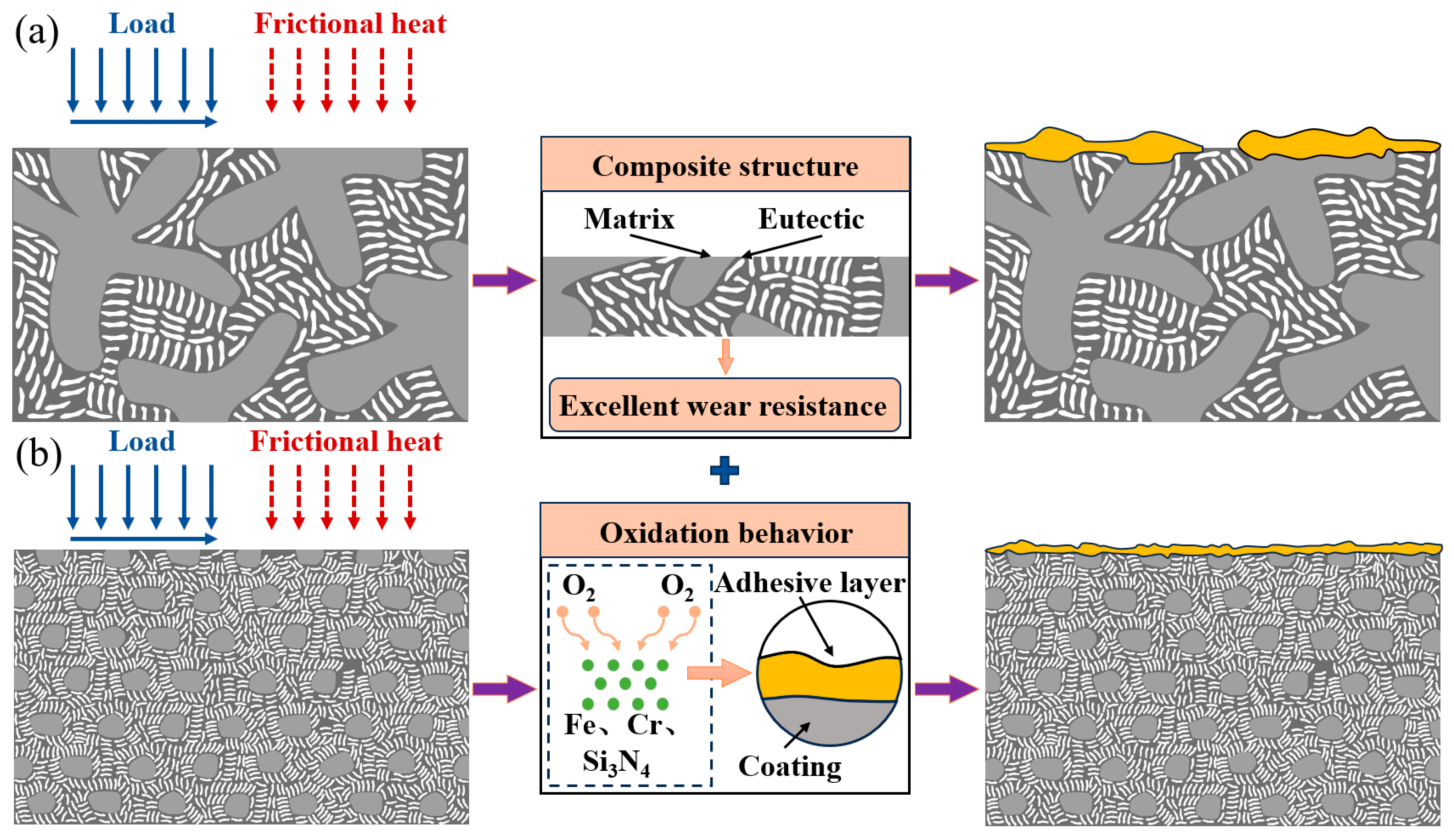
Wear-resistant mechanism of the coatings fabricated by different technologies: (**a**) LIC, (**b**) EHIC.

Figure 14 illustrates the wear mechanisms of Fe-Cr-B coatings fabricated by LIC and EHIC. At the macroscopic level, coatings produced by both methods exhibit high microhardness, which confers strong resistance to plastic deformation, thereby enabling effective resistance to indentation by the abrasive counterface and ploughing by surface asperities. At the microscopic level, the eutectic regions exhibit high nano-hardness, a high elastic modulus, and high elastic recovery. These characteristics provide essential longitudinal load support and lateral resistance to ploughing, thereby establishing the microstructural foundation for the wear performance of the coating.

Oxidation induced by frictional heating during wear significantly affects the wear resistance of coating. The oxidation of Fe and Cr, particularly from the matrix and from regions adjacent to the M_2_B phase in the eutectic structure, weakens the structural support for the hard M_2_B phase. Consequently, the ability of coating to resist indentation and ploughing is diminished. Simultaneously, oxidation products and wear debris undergo a cyclic process of detachment and compaction, forming a tribolayer on the surface. This layer can reduce direct contact between the Si_3_N_4_ ball and the coating, thereby alleviating wear. However, if this tribolayer or underlying M_2_B particles detach, they may enter the contact zone as abrasives, thereby accelerating material loss.

The disparity in mechanical properties between distinct microstructural regions results in pronounced selective wear during dry friction. Softer regions, including the matrix and interphase region within the eutectic structure, undergo preferential wear. In contrast, the hard M_2_B phase serves as the primary load-bearing and wear-resistant constituent. The LIC coating contains a high fraction of coarse matrix, as shown in Figure 14a. This coarse microstructure exacerbates wear non-uniformity, weakens support for the M_2_B phase, and accelerates overall material loss and oxidation. As wear progresses, an adhered tribolayer on the wear scar surface can temporarily mitigate material loss. However, extensive detachment of this layer exposes the underlying coating to direct contact with hard abrasive particles, such as M_2_B or Si_3_N_4_. This contact induces severe abrasive wear, thereby resulting in a significant increase in material loss for the LIC coating.

For the EHIC coating, the high fraction of eutectic regions offers sustained wear protection throughout the testing process. As shown in Figure 14b, microstructural refinement and improved homogeneity during the early stages of wear promote more uniform material removal, thereby mitigating the localized severity of selective wear. The tribolayer, working in concert with the eutectic region, effectively suppresses the vertical progression of wear damage. Even if the tribolayer detaches in later stages, the high fraction of wear-resistant eutectic structure retains its protective capacity.

### 4.2. Tribocorrosion Behavior of Samples

The tribocorrosion and dry friction behaviors of the Fe-Cr-B coating differ substantially. The wear rate under tribocorrosion conditions is significantly higher than that observed during dry friction. Material loss during tribocorrosion arises from a synergistic interaction between corrosion and mechanical wear [36].

In the corrosive environment, anodic dissolution at the interfaces between the matrix and M_2_B phase weakens interfacial cohesion. As a result, the M_2_B phase becomes more susceptible to detachment under shear stress. Once detached, these hard particles act as third-body abrasives, ploughing deep furrows into the surface and thereby substantially increasing material removal. Concurrently, electrochemical corrosion leads to general surface softening, thereby reducing the resistance of the coating to micro-cutting. Furthermore, the liquid medium inhibits the formation of a protective tribolayer, thereby exacerbating abrasive and micro-cutting effects and leading to increased material loss.

Although XPS analysis confirmed the presence of corrosion products on the worn surface, these products did not reduce the wear rate of coating. This is because the ongoing wear process continuously disrupts and removes surface passivation films or corrosion layers, repeatedly exposing fresh material to the corrosive medium and thereby accelerating electrochemical corrosion [31]. Furthermore, the groove structures generated by abrasive ploughing create sites for intensified anodic dissolution. Consequently, under this synergistic tribocorrosion effect, the wear rate of the coating is significantly higher than that observed under dry friction.

The difficulty in forming a stable and protective tribolayer allows the Si_3_N_4_ ball to maintain direct contact with the coating. Under such conditions, the wear rate becomes largely dependent on the fraction of softer matrix regions. Consequently, the LIC coating, with its high proportion of these regions, experiences a higher wear rate. Under applied normal load, the Si_3_N_4_ ball indents the softened surface. The general softening induced by anodic dissolution, combined with coarse microstructure, facilitates the embedding and abrasive action of hard particles. This leads to severe abrasive wear, producing deep and wide furrows and substantially increasing material removal.

In contrast, the EHIC coating exhibits a high fraction of eutectic region and a refined, uniform microstructure, conferring upon it superior wear resistance. Even in the absence of a protective tribolayer, the M_2_B-rich eutectic structure effectively resists plastic deformation and micro-cutting. The fine, densely distributed M_2_B phases act as persistent barriers along the wear path, continuously impeding the penetration and cutting action of hard abrasives, thereby significantly enhancing wear resistance. Simultaneously, the reduced fraction and size of the susceptible matrix limit their exposure to the corrosive environment, thereby mitigating their accelerated wear by abrasive particles. As a result, only shallow ploughing marks are observed on the worn EHIC surface.

In summary, the eutectic region is the key determinant of wear resistance in Fe-Cr-B coating. This microstructure provides continuous, uniform, and effective protection, constituting the fundamental reason for the excellent wear performance.

## 5. Conclusions

This study investigated the microstructure, dry friction, and tribocorrosion behaviour of Fe-Cr-B coatings fabricated by LIC and EHIC. The key findings are summarized as follows:

(1) The microstructure of Fe-Cr-B coatings fabricated by LIC and EHIC consists of a matrix region and a eutectic region comprising M_2_B and martensite. The matrix is enriched in Fe and C, whereas the eutectic region is enriched in Cr and B. As the scanning speed increases, the columnar matrix transforms into an equiaxed structure, and the overall microstructure becomes significantly refined.
(2) The hardness of Fe-Cr-B coatings produced by LIC and EHIC remains stable within the range of 782.3 to 796.1 HV, providing a favourable basis for wear resistance. The dry sliding wear mechanism involves oxidative wear and abrasive wear. The eutectic region serves as the primary load-bearing and wear-resistant constituent within the coating. Benefiting from an increased fraction of the eutectic region, the dry sliding wear performance is enhanced. Furthermore, a tribolayer formed during testing isolates direct contact between the Si_3_N_4_ and the coating, further improving wear resistance. Consequently, as the scanning speed increases, the wear rate decreases from99.7 × 10^−3^ mm^3^·N^−1^·m^−1^ to 50.2 × 10^−3^ mm^3^·N^−1^·m^−1^.
(3) In corrosive environments, the difficulty in forming a stable tribolayer, combined with electrochemical corrosion, weakens the matrix and phase interfaces, thereby increasing the susceptibility of the coating to micro-cutting and material detachment. The tribocorrosion mechanism involves both oxidation and abrasion, with abrasive wear becoming the dominant mode. Owing to the higher fraction of the eutectic region, the EHIC coating maintains superior wear performance, with its wear rate decreasing from 255.8 × 10^−3^ mm^3^·N^−1^·m^−1^ to 175.4 × 10^−3^ mm^3^·N^−1^·m^−1^ as the scanning speed increases.

## Figures and Tables

**Table 1:** The optimal process parameters of TLC and EHIC experiments in this study.

Sample		Laser Power (W)	Scanning Speed (m/min)	Laser Spot Diameter (mm)	Overlap Rate	Powder Flow Rate (g/min)
TLC	1 m/min-300 °C	2200	1	3	50%	8
EHIC1	10 m/min-300 °C	3200	10	3	70%	15
EHIC2	20 m/min-300 °C	3800	20	3	80%	20
EHIC3	40 m/min-300 °C	4800	40	3	90%	25

**Table 2:** The wear characteristics of LIC and EHIC fabricated Fe-Cr-B alloys.

Coating	Maximum Wear Depth (μm)	Maximum Wear Width (μm)	Wear (10^−3^ mm^3^)	Wear Rate (10^−3^ mm^3^·N^−1^·m^−1^)
1 m/min-300 °C	10.5 ± 0.4	912.7 ± 50.4	14.6 ± 0.8	99.7 ± 5.5
10 m/min-300 °C	8.9 ± 0.3	845.7 ± 46.9	12.4 ± 0.4	82.4 ± 2.6
20 m/min-300 °C	7.5 ± 0.3	820.2 ± 16.1	10.3 ± 0.3	68.9 ± 2.3
40 m/min-300 °C	5.6 ± 0.3	790.7 ± 38.3	7.5 ± 0.9	50.2 ± 6.1

**Table 3:** The tribocorrosion characteristics of LIC and EHIC fabricated Fe-Cr-B alloys.

Coating	Maximum Wear Depth (μm)	Maximum Wear Width (μm)	Wear (10^−3^ mm^3^)	Wear Rate (10^−3^ mm^3^·N^−1^·m^−1^)
1 m/min-300 °C	14.9 ± 0.2	1133.2 ± 37.8	38.4 ± 5.7	255.8 ± 38.2
10 m/min-300 °C	14.6 ± 0.2	1121.1 ± 26.5	34.7 ± 7.7	231.1 ± 51.6
20 m/min-300 °C	12.8 ± 0.2	1084.5 ± 14.2	32.3 ± 4.6	215.4 ± 30.8
40 m/min-300 °C	11.8 ± 0.3	1064.6 ± 30.9	26.3 ± 4.1	175.4 ± 27.1

**Table 4:** Mechanical parameters of different phases.

Region	Coating	H (Gpa)	E (Gpa)	H/E	H^3^/E^2^ (Gpa)
Matrix	1 m/min-300 °C	10.14	285.13	0.0356	0.0128
	10 m/min-300 °C	10.69	288.03	0.0371	0.0147
	20 m/min-300 °C	10.94	291.19	0.0376	0.0154
	40 m/min-300 °C	11.75	304.22	0.0386	0.0175
Eutectic	1 m/min-300 °C	12.04	314.85	0.0382	0.0176
	10 m/min-300 °C	13.01	319.49	0.0407	0.0216
	20 m/min-300 °C	13.11	321.74	0.0407	0.0218
	40 m/min-300 °C	13.99	330.33	0.0424	0.0251

## Data Availability

The original contributions presented in this study are included in the article. Further inquiries can be directed to the corresponding author.
